# Supplementary material for: Rectal Sensory and Compliance Testing: A Method Comparison Study between High-Resolution Anorectal Manometry and Barostat Investigations
Source: Diagnostics (Basel). 2024 Feb 6;14(4):351. doi: 10.3390/diagnostics14040351 (PMC10888286; doi:10.3390/diagnostics14040351)
Supplement: Supplementary file 1 [file diagnostics-14-00351-s001.zip › diagnostics-2829123-supplementary.pdf]

Supplemental Table S1. A stepwise approach and checklist to the design, data analysis and data interpretation of rectal compliance and sensibility method comparison studies.

Study phase	Topic	Checklist item
Design	Measurement protocols	• Standardized testing protocol and the London classification for disorders of anorectal function<br>• Phenotyping of subjects for large-scale studies on patients with IBS<br>• Create a protocol for the measurement of rectal compliance with HRAM based on available data and variables used in barostat studies to allow comparison.
	Criteria for agreement	• A correlation factor above 0.6 between variables will then be assessed using the Bland–Altman method of measuring agreement.<br>• There are no data regarding acceptable bias or level of agreement, and intraindividual variation needs to be taken into account. A bias or confidence interval approaching the observed median values of the respective variables should be regarded as a poor level of agreement.
	Sample size	Our study involved the assessment of rectal function in 26 participants. Power calculations are dependent on the expected difference, which is unknown.<br>• Power calculation using a clinically relevant difference of 20 mmHg between the same sensory thresholds suggested that 11 participants might be sufficient.
	Reference technique	The rectal barostat investigation is considered the gold standard of rectal sensory and compliance testing.
Data analysis	Normal distribution	• We will assume that the results are not normally distributed.<br>• The between-method differences will be checked for normality since this allows the use of parametric methods to obtain confidence intervals.

Study phase	Topic	Checklist item
	Bland–Altman analysis	Calculate the bias, level of agreement and their corresponding 95% confidence intervals.
		All participants are investigated once with each method.
		Check the presence of proportional bias, spread or both visually in the Bland–Altman plot and the Pearson correlation test between the between-method differences and means.
Interpretation	Reference precision	Available studies indicate some intraindividual variability in barostat studies.<br>Repeated HRAM investigations in a subgroup of the cohort will aid in interpreting bias and the level of agreement.
